# Supplementary figures and images for: Comparative analysis of virulence determinants, phylogroups, and antibiotic susceptibility patterns of typical versus atypical Enteroaggregative E. coli in India
Source: PLoS Negl Trop Dis. 2020 Nov 18;14(11):e0008769. doi: 10.1371/journal.pntd.0008769 (PMC7673547; doi:10.1371/journal.pntd.0008769)

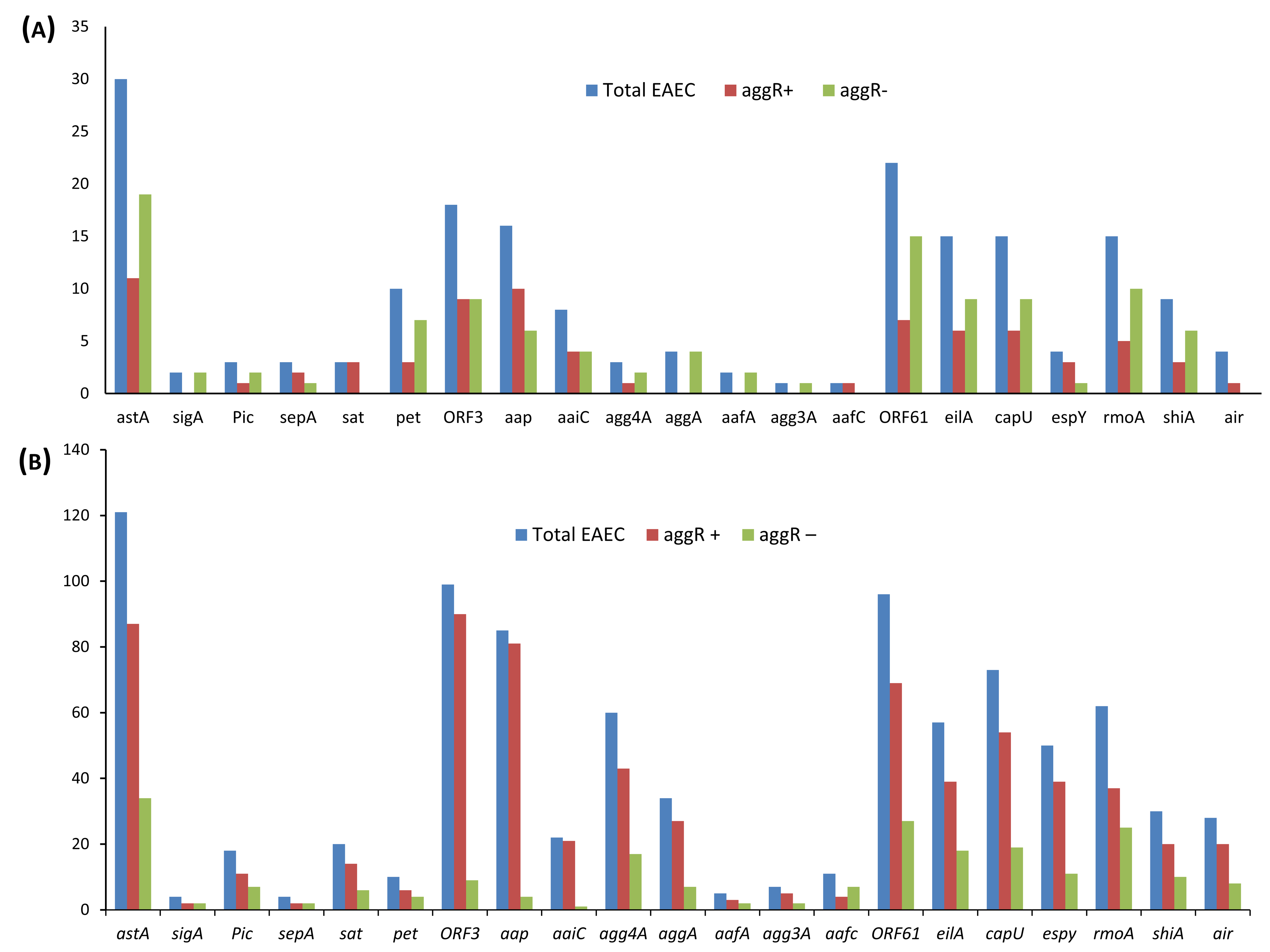

Supplement: S1 Fig — Distribution of virulence-related markers among tEAEC and aEAEC in control A) and diarrheal group B). tEAEC: typical enteroaggregative E. coli, aEAEC: atypical enteroaggregative E. coli. (TIF) [file pntd.0008769.s001.tif]
