# Supplementary material for: Comparative analysis of virulence determinants, phylogroups, and antibiotic susceptibility patterns of typical versus atypical Enteroaggregative E. coli in India
Source: PLoS Negl Trop Dis. 2020 Nov 18;14(11):e0008769. doi: 10.1371/journal.pntd.0008769 (PMC7673547; doi:10.1371/journal.pntd.0008769)
Supplement: S1 Table — (DOCX) [file pntd.0008769.s002.docx]

**S1 Table.** Region-wise severity score

| **Centre name** | **Total sample** | **Severe n=583(%)** | **Moderate n=363 (%)** | **Mild (%) n=265** | ***P*-value** |
| --- | --- | --- | --- | --- | --- |
| Chandigarh | 494 | 211 (42.1%) | 125 (25.0%) | 158 (31.9%) | 0.0001* |
| Punjab | 247 | 131 (53%) | 84 (34.0%) | 32 (13.0%) | 0.0001* |
| Haryana | 221 | 114 (51.8%) | 79 (35 %) | 28 (12.6%) | 0.0002* |
| Uttarakhand | 114 | 62 (53.4%) | 36 (31%) | 17 (15.5%) | 0.05* |
| Himachal Pradesh | 133 | 65 (48.8%) | 39 (29.3%) | 29 (21.8%) | 0.0001* |
| Grand Total | 1210 | 583 (48.1%) | 363 (30.0%) | 265 (21.8%) | 0.0001* |

*Statistically significant (P<0.05) when severe and moderate severity was pooled and compared with mild severity. Data was analysed by using Fischer’s exact test.
