# Supplementary material for: Comparative analysis of virulence determinants, phylogroups, and antibiotic susceptibility patterns of typical versus atypical Enteroaggregative E. coli in India
Source: PLoS Negl Trop Dis. 2020 Nov 18;14(11):e0008769. doi: 10.1371/journal.pntd.0008769 (PMC7673547; doi:10.1371/journal.pntd.0008769)
Supplement: S2 Table — (DOCX) [file pntd.0008769.s003.docx]

**S2 Table.** Distribution of EAEC virulence related markers among in diarrheal and control group.

| **EAEC factor** | **Diarrheal group** | **Control group** | **Total n=171 (%)** | **Odds Ratio** | **[95% CI]** | **Z-value** | ***P-*Value** |
| --- | --- | --- | --- | --- | --- | --- | --- |
|  | **Total (%) n=138** | **Total (%) n=33** |  |  |  |  |  |
| *ast*A | 121 (87.6) | 30 (90.9) | 151 | 0.71 | 0.1958 to 2.5879 | 0.51 | 0.6 |
| *sig*A | 4 (2.8) | 2 (6.0) | 6 | 0.46 | 0.0811 to 2.6408 | 0.86 | 0.38 |
| *Pic* | 18 (13.0) | 3 (9.0) | 21 | 1.5 | 0.4145 to 5.4279 | 0.61 | 0.53 |
| *sep*A | 14 ( 10.14) | 3 (9.0) | 17 | 1.12 | 0.3049 to 4.1810 | 0.182 | 0.8 |
| *sat* | 20 (14.5) | 3 (9.0) | 23 | 1.69 | 0.4722 to 6.0837 | 0.809 | 0.41 |
| *pet* | 10 (7.24) | 10 (30.3) | 20 | 0.17 | 0.0673 to 0.4800 | 3.42 | 0.0006* |
| ORF3 | 99 (71.8) | 18 (54.5) | 117 | 2.11 | 0.9707 to 4.6100 | 1.88 | 0.05* |
| *aap* | 85 (61.6) | 16 (48.4) | 101 | 1.7 | 0.7937 to 3.6584 | 1.36 | 0.17 |
| *aai*C | 22 (16.4) | 8 (24.2) | 30 | 0.5 | 0.2368 to 1.4833 | 1.18 | 0.26 |
| *agg4*A | 60 (43.4) | 3 (9.0) | 63 | 7.6 | 2.2402 to 26.4131 | 3.24 | 0.0012* |
| *agg*A | 34 (24.6 | 4 (12.1) | 38 | 2.37 | 0.7774 to 7.2267 | 1.517 | 0.1292 |
| *aafA* | 5 (3.6) | 2 (6.0) | 7 | 0.58 | 0.1080 to 3.1447 | 0.628 | 0.53 |
| *agg3A* | 07 (5.07) | 1 (3.0) | 8 | 1.7 | 0.2031 to 14.3985 | 0.49 | 0.62 |
| *aafc* | 11 (7.9) | 1 (3.0) | 12 | 2.56 | 0.3200 to 20.6282 | 0.88 | 0.37 |
| ORF61 | 96 (69.5) | 22 (66.6) | 118 | 0.64 | 0.2959 to 1.4217 | 1.081 | 0.27 |
| *eil*A | 57 (41.3) | 15 (45.4) | 72 | 0.84 | 0.3932 to 1.8137 | 0.43 | 0.66 |
| *cap*U | 73 (52.8) | 15 (45.4) | 88 | 1.34 | 0.6288 to 2.8887 | 0.767 | 0.44 |
| *espy* | 50 (36.2) | 4 (12.12) | 54 | 4.11 | 1.3691 to 12.3940 | 2.51 | 0.011* |
| *rmo*A | 62 (44.9) | 15 (45.4) | 77 | 0.70 | 0.3304 to 1.4911 | 0.921 | 0.35 |
| *shi*A | 30 (21.7) | 9 (27.2) | 39 | 0.74 | 0.3115 to 1.7616 | 0.67 | 0.49 |
| *air* | 28 (20.) | 4 (12.12) | 32 | 1.84 | 0.5993 to 5.6828 | 1.068 | 0.28 |

*Statistically significant (P<0.05) when virulence genes from diarrheal and control groups were compared. Data was analysed by using Fischer’s exact test.
