# Supplementary material for: Comparative analysis of virulence determinants, phylogroups, and antibiotic susceptibility patterns of typical versus atypical Enteroaggregative E. coli in India
Source: PLoS Negl Trop Dis. 2020 Nov 18;14(11):e0008769. doi: 10.1371/journal.pntd.0008769 (PMC7673547; doi:10.1371/journal.pntd.0008769)
Supplement: S3 Table — (DOCX) [file pntd.0008769.s004.docx]

**S3 Table.** Combination of virulence markers among diarrheal and control EAEC strains

| **EAEC (No. of strains)** | **Gene combination** | **No. (%) of strains** |
| --- | --- | --- |
| **Diarrhea (138)** | *ast*A*,* ORF3*, aap, agg*R*, ,*ORF61*, cap*U | 37 (26.8) |
|  | ORF3,*aap, agg*R*, ,*ORF61*, cap*U*,* | 3 (2.17) |
|  | *ast*A*,* ORF3*, agg*R*, ,*ORF61*, cap*U*,* | 5 (3.6) |
|  | *ast*A*,* ORF3*, aap, agg*R*, cap*U | 7 (5.07) |
|  | *ast*A*,* ORF3*, aap, agg*R*,* ORF61 | 18 (13.04) |
|  | ORF3,*aap, agg*R*, cap*U | 1 (0.7) |
|  | *ast*A*, aap, agg*R*, cap*U | 1 (0.7) |
|  | *ast*A*,* ORF3*,* ORF61*, capU* | 3 (2.17) |
|  | *ast*A*, ORF3, aggR,* ORF61 | 6 (4.34) |
|  | *ast*A*,* ORF3,*aap, ORF61* | 1 (0.7) |
|  | *ast*A*,* ORF3*, aap, agg*R | 11 (7.97) |
|  | ORF3*, aap, aggR* | 1 (0.7) |
|  | *astA,* ORF3*, cap*U | 1 (0.7) |
|  | *astA, aap, cap*U | 1 (0.7) |
|  | ORF61*, cap*U | 2 (1.44) |
|  | *ast*A*, cap*U | 2 (1.44) |
|  | *ast*A*,* ORF61 | 5 (3.6) |
|  | *ast*A*, aap* | 2 (1.44) |
|  | *ast*A*,*ORF3 | 1 (0.7) |
|  | *cap*U | 1 (.7) |
|  | ORF61 | 5 (3.6) |
|  | *ast*A | 3 (2.17) |
|  | *none* | 6 (4.34) |
| **Control (33)** | *ast*A*,* ORF3,*aap, agg*R*, ,*ORF61*, cap*U*,* | 3 (9.09) |
|  | *ast*A*, aap, agg*R*, ,*ORF61*, cap*U*,* | 1 (3.03) |
|  | *ast*A*,* ORF3*, agg*R*, ,*ORF61*, cap*U*,* | 1 (3.03) |
|  | *ast*A*,* ORF3,*aap,* ORF61*, cap*U | 2 (6.06) |
|  | *ast*A*,* ORF3*, aap, agg*R*,* ORF61 | 3 (9.09) |
|  | *ast*A*, aap, agg*R*, cap*U | 1 (3.03) |
|  | *astA,* ORF3*,* ORF61*, capU* | 2 (6.06) |
|  | *ast*A*,* ORF3*, aap, agg*R | 2 (6.06) |
|  | *ast*A*,* ORF61*, cap*U | 4 (12.12) |
|  | *ast*A*,* ORF3*,* ORF61 | 3 (9.09) |
|  | *astA,*ORF3*,aap* | 1 (3.03) |
|  | *ORF61, capU* | 1 (3.03) |
|  | ORF3*,* ORF61 | 1 (30.3) |
|  | *ast*A*, cap*U | 1 (30.3) |
|  | *ast*A*, aap* | 4 (12.12) |
|  | *ast*A | 1 (3.03) |
