# Supplementary material for: Comparative analysis of virulence determinants, phylogroups, and antibiotic susceptibility patterns of typical versus atypical Enteroaggregative E. coli in India
Source: PLoS Negl Trop Dis. 2020 Nov 18;14(11):e0008769. doi: 10.1371/journal.pntd.0008769 (PMC7673547; doi:10.1371/journal.pntd.0008769)
Supplement: Attachment — Submitted filename: Response to reviewers.docx [file pntd.0008769.s005.docx]

**Response to Reviewers:**

**Reviewer #1:**

**Question 1**. Lines 176-182: More detailed methods need to be provided for the selective culturing and identification of the *E. coli*, and other diarrheal pathogens described.

**Answer:** As per suggestion method of selective culturing and identification of the *E. coli* and other diarrheal pathogens is explained in detail in the revised manuscript and reference has been provided (Ref. 30) (please refer lines 191-213).

**Reviewer #2:**

**Reviewer #3**:

The objectives are clearly stated, and the study design is appropriate.

**Question 2**. For the population studied in the control group: did the authors follow up if the children that tested positive showed or had previously shown any symptoms? Does infection in the control group sample mean the presence of bacteria or the presence of symptoms? How long after or before the symptoms set in can the bacteria be detected in the stool sample?

**Answer:** We followed the children in the control group that tested positive for DEC, and none of them showed or had previously any symptoms among them. The infection in the control group means only the presence of bacteria without any symptoms.

**Question 3.** Are the antibiotics tested here the first line of treatment given to children showing symptoms?

**Answer:** No, only ciprofloxacin, co-trimoxazole, and ceftriaxone are clinically used. We tested other antibiotics for epidemiological surveillance of antimicrobial resistance (AMR) as being carried out for all diarrheal pathogens in our laboratory.

**Reviewer #1:**

**Question 4.**Fig 1: How are the sampling sites indicated? Please describe.

**Answer:** Sampling sites indicated in figure 1 are the regional health centers of different cities across the North India region [Chandigarh (PGIMER), Civil hospital Manimajra; Haryana (Civil hospital Panchkula and Civil hospital Ambala Cantt); Himachal Pradesh (Government Medical College (GMC) Hamirpur, Indira Gandhi Medical College (IGMC) Shimla, and Rajendra Prasad Government Medical College Kangra (RPGMC) Tanda); Punjab (Civil hospital and private laboratory Mohali and Private laboratory Ludhiana); and Uttarakhand (All India Institute of Medical Science (AIIMS) Rishikesh, District hospital Rudrapur and District hospital Haridwar)] from where stool samples of diarrheal and healthy children were collected thus representing a large geographical area. The Map link has been attached, which can be opened by pressing CTRL+ click. Clicking on each red dot on the map shows the name of the place and number of samples collected there (please refer to fig 1 and line 180-189).

**Question 5.**Table 1: Are these the primers that were used for pathotype classification and are described elsewhere in the manuscript as M-PCR? Please clarify.

**Answer:** Detail of primers used to classify pathotypes has been described in the material and methods section of the manuscript (please refer to table 1 and line 209-213).

**Question 6**. Fig 3: Please provide greater detail in the figure legend, describing what is in each lane. The blue arrows stretching across the image are difficult to see, and some of the words in red next to the gel are smaller and difficult to read.

**Answer:** As per suggestion, figure 3 has been formatted and replaced, and detail of each lane has been described in the figure legend (please refer to fig 3 and lines 289-293).

.

**Question 7.**Table 9: These are both tEAEC and aEAEC?

**Answer:** No, table 9 is containing only EAEC strains isolated from diarrheal and control group. Now, this table has been removed from the revised manuscript and supplied as supplementary material (please refer to S2 table of supplementary data).

**Question 8.**Tables 9, 10, and 11: These tables could be combined to save space in the manuscript.

**Answer:** Table 9 is containing only EAEC strains isolated from the diarrheal and control group, whereas table 10 and 11 describing tEAEC and aEAEC from the diarrheal and control group, respectively. So, it is difficult to merge table 9,10, and 11. However, as per the suggestion of reviewer 2 table 10 and 11 has been combined in the revised manuscript as table 8. Table 9 has been removed from the revised manuscript and supplied as supplementary material (please refer to S2 table of supplementary data).

**Question 9.**Table 12: This table could be moved to supplemental data.

**Answer:** As per suggestion, Table 12 is removed in the revised manuscript and supplied as a supplementary file (please refer to S3 table of supplementary data).

**Question 10.**Figures 6 and 7: These figures could be combined to make it easier to compare virulence gene trends between diarrheal vs. control EAEC.

**Answer:** As per suggestion, Figures 6 and 7 have been combined in the revised manuscript and shifted to supplementary data as S1 Fig in supplementary data.

**Question 11.**Tables 13, 14, and 15: These tables should be combined for ease of comparison.

**Answer:** Suggestion has been incorporated in the manuscript. Tables 13, 14, and 15 has been combined and updated in the revised manuscript as table 9.

**Question 12.**Figure 8: This figure could be removed as it is redundant with the tables

**Answer:** As per suggestion, figure 8 has been removed in the revised manuscript.

**Question 13**. Tables 16, 17, and 18: These tables should be combined for ease of comparison. Also, it’s not clear why Table 16 has colors.

**Answer:** As per suggestion, Tables 16, 17, and 18 has been combined in the revised manuscript as table 10, and the format of the table has been changed; there are no colors now.

**Reviewer #2:**

**Question 14.** A number of figures are duplicating the information given in the Tables. This should be avoided

**Answer:** As per suggestions, duplicate tables and figure are either removed, combined or supplied as supplementary material in the revised manuscript. Table 6, 9, and 12 has been shifted to supplementary data as S1, S2, and S3 table respectively in the revised manuscript. Table (10, 11), table (13, 14, 15), and table (16, 17, 18) has been combined as table 8, table 9 and table 10, respectively in the revised manuscript. Fig 4, 5 and 8 has been removed, and fig 6 and 7 has been combined and shifted as supplementary data as S1 fig in the revised manuscript.

**Question 15.** Fig 2 shows the information on 273 DEC positive children. Hence, the bar showing DEC strains may be omitted since the total number of DEC can be made out from the other bars, which depict subsets of DEC.

**Answer:** As per the suggestion bar showing DEC strains has been removed in the revised manuscript.

**Question 16.** Table 3- The percentage of EAEC etc., has been calculated out of 273 DEC, not the total number of patients i.e.1210. However, in the break up of ages of patients, the total has been calculated out of the total number of patients. A uniform method should be used in the table.

**Answer:** As per suggestion, the percentage of EAEC and other pathotypes has been calculated from the total number of patients.

**Question 17.** Fig 4 is not clear as the legend says, "Distribution of DEC groups by age in children without diarrhea". However, the ages cannot be made out in the figure. Since the same information is seen in Table 4, Fig 4 can be omitted.

**Answer:** As per suggestion, Fig 4 has been removed in the revised manuscript.

**Question 18**. Table 5 gives "Distribution of DEC pathotypes among diarrheal and healthy children". Hence the total number of patients need not be mentioned in the Table. This confuses the reader.

**Answer:** As per suggestion, the total number of patients has been removed from the table, and the percentage of EAEC, EPEC, and ETEC is calculated from the total number of DEC (273) (please refer to table 5).

**Question 19.** Fig 5 is not needed. The information given in the text is sufficient.

**Answer:** As per suggestion, Fig 5 has been removed in the revised manuscript.

**Question 20.** Fig 6 is not needed. Table 10 gives the same information. Table 10 and 11 can be combined for better comparison.

**Answer:** As per suggestion of reviewer 1,figures 6 and 7 have been combined to compare virulence gene trends between diarrheal vs. control EAEC and has been shifted to supplementary data (please refer to S1 fig of supplementary data). Table 10 and 11 has been combined in the revised manuscript as table 8.

**Question 21.** Similarly, Fig 7 is repetitive of Table 11.

**Answer:** As per suggestion to avoid repetition, figure 7 is combined with figure 6 as S1 fig (please refer supplementary data), and table 10 is combined with table 11 as table 8 in the revised manuscript.

**Question 22.** Fig 8 is repetitive.

**Answer:** As per suggestion, Fig 8 has been removed in the revised manuscript.

**Question 23**. Fig 14 and 15 can be combined.

**Answer:** As there is no fig 14 and 15, but as per suggestion, table 14 and 15 is combined in the revised manuscript as table 9 (please refer to table 9).

.

**Reviewer #3:**

**Question 24.**Table 3: The data is distributed 0-2, 2-5 and <5. Have the authors analyzed if 0-2 and 2-5 are statistically different, before pooling in the data for analysis.

Answer: Yes, we have compared all groups, but we found them significant only when populations of 0-1 1-2 and 2-5 years were pooled and analyzed with children aged 5-10 years (please refer to table 3).

**Question 25.** Table 4: The groups for age distribution should be kept the same for comparable analysis.

**Answer:** In the diarrheal group, stool samples were collected from age <10 years of children, and in the control group, stool samples were collected from children aged <5 years. So, as per suggestion in the diarrheal group, we have categorized children into 0-1, 1-2, 2-5 and 5-10 years of age group whereas in the control group age group are divided into 0-1,1-2 and 2-5 years of age group. In table 4 we have compared the pooled prevalence of DEC in children aged 0-1 and 1-2 years to children aged 2-5 years. In table 3, children of 0-1 1-2 and 2-5 years were pooled and analyzed with children aged 5-10 years (please refer to table 3 and 4).

**Question 26.** Table 5: The authors need to show data only from DEC positive samples and not overall samples. Please carry out statistical analysis for EAEC positive samples in the control vs diseases group.

**Answer:** As per suggestion, the total number of patients has been removed from the table, and the percentage of EAEC, EPEC, and ETEC is calculated from the total number of DEC (273) (please refer to table 5).

**Question 27.**Table 8: is tEAEC and aEAEC significant in either diarrheal or the control group?

**Answer:** As per the query, we have compared tEAEC and aEAEC in either diarrheal and control group and found statistically significant and the same is incorporated in the revised manuscript (please refer to table 7).

Reviewer #3:

**Question 28.**All the figures and legends require more description.

**Answer:** As per suggestion, all the figure legend has been described in detail in the revised manuscript.

**Question 29.**Fig 2,4: Do not add DEC data as a bar in the graph as the authors are showing the distribution of subDEC strains.

**Answer:** As per suggestion DEC bar from fig 2 has been removed in the revised manuscript (please refer fig 2) whereas fig 4 has been removed from the revised manuscript.

**Question 30.**Table 6: can be removed or moved to supplements as it discusses the severity of non-DEC positive infections.

**Answer:** As per suggestion, Table 6 is removed in the revised manuscript and supplied as a supplementary file (please refer to S1 table of supplementary data).

**Question 31.**Table 9, 10, 11, 12, and its associated figures need to be condensed and represented differently, focusing on major findings.

**Answer:** As per suggestion Table 10 and 11 has been combined as table 8. Table 9, 12 has been provided as a supplementary S2 and S3 table in the revised manuscript.

**Question 32.**Table 13, 14, 15 and fig 8: also needs to be represented differently in a condensed format.

**Answer:** As per suggestion, Table 13, 14, 15 are combined as table 9, and to avoid repetition fig 8 has been removed in the revised manuscript.

**Question 33.**Table 16, 17, 18 also needs to be condensed and needs removal of redundant data.

**Answer:** As per suggestion, Table 16, 17, 18 are combined in the revised manuscript as table 10.

**Reviewer #1:** The manuscript titled “Comparative analysis of virulence determinants, phylogroups and antibiotic susceptibility patterns of typical versus atypical Enteroaggregative E. coli in India” describes the prevalence of EAEC compared to other diarrheagenic E. coli pathotypes among diarrheal versus non-diarrheal stools of children in North India. The authors further described the EAEC isolates by classifying them as tEAEC or aEAEC and comparing the virulence gene content of these sub-groups of EAEC among the diarrheal versus non-diarrheal stools.

Throughout the manuscript, there are missing spaces between words.

Missing spaces have been corrected.

**Question 34.**Lines 71-72: Please describe what the CVD432 probe is.

**Answer:** CVD432 probe is a DNA probe from the pAA plasmid of EAEC, has been reported to be specific for the detection of EAEC. CVD432 probe has been described in the revised manuscript (please refer lines 69-71).

**Question 35**. Line 75: Please write out PGI.

**Answer:** Postgraduate Institute of Medical Education and Research (PGIMER) is a medical and research institution in [Chandigarh](https://en.wikipedia.org/wiki/Chandigarh), [India](https://en.wikipedia.org/wiki/India). It is the leading tertiary care hospital of the region and caters to patients from all over  [Punjab](https://en.wikipedia.org/wiki/Punjab,_India), Jammu and Kashmir, [Himachal Pradesh](https://en.wikipedia.org/wiki/Himachal_Pradesh) and [Haryana](https://en.wikipedia.org/wiki/Haryana) (please refer lines 74-79).

**Question 36.** Lines 77-78: Was gene expression examined? Or were the genes detected using PCR?

**Answer:** No, gene expression was not examined. The presence of virulence genes were detected by using PCR (please refer lines 81-82).

**Question 37.** Lines 79-81: It is not clear what is meant by “our signature sequence”. Are you referring to the combination of the three genes astA, aap, and aggR. Are you saying the detection of these genes can be used to identify both tEAEC and aEAEC? This is not clear.

**Answer:** “Our signature sequence” refers to the combination of the three genes *ast*A, *aap*, and *agg*R, which is present in more than 50% of EAEC strains. Hence we suggested the combination of three genes (*ast*A, *aap*, and *agg*R) would be ideal as probes for detection of EAEC as well as both tEAEC and aEAEC (please refer lines 83-85).

**Question 38.**Line 81: Write out MDR, and clarify what is meant by “more resistant”. Do the tEAEC typically exhibit resistance to a greater number of antibiotics? Is there an average number of antibiotics that the tEAEC exhibited reduced susceptibility to compared to the aEAEC?

Answer: The multidrug resistance (MDR), defined as acquired resistance toward three or more antibiotics from different antibiotic classes tested (please refer to material and method section; lines 260-261). In our findings, we observed that 75.8% of the EAEC strains were MDR. This line has been updated in the revised manuscript (please refer lines 85-87). In line 81 “more resistant” means the percentage of tEAEC strains is higher than the percentage of aEAEC strains (please refer lines 85-87). Yes, we found that tEAEC exhibits resistance to a greater number of antibiotics with respect to aEAEC (please refer lines 85-87).

**Question 39.** Lines 108-109: Please clarify the sentence “Clinical features of EAEC diarrhea are usually well-defined in sporadic cases outbreaks and voluntary model.”. It is not clear what is meant by sporadic cases. Or what is meant by voluntary model. Also, please describe the clinical features.

**Answer:** In this sentence, clinical features refers to the symptoms like watery, mucoid, low-grade secretory diarrhea, grossly bloody stools, and little to no vomiting.  Sporadic cases refer to an occasional or irregular reported infection, whereas the volunteer model refers to a controlled human infection model where a volunteer is infected with a pathogen as a part of a tightly controlled experiment under ethical guidelines (please refer lines 113-116).

**Question 40.** Lines 133-136: The revised classification scheme by Jaureguy et al. (PMID: 19036134) described 5 lineages of E. coli (A+B1, B2, D, E, and F). The statement that most DEC strain belongs to phylogroup D, while commensals belong to phylogroups A and B1 is not correct. ETEC in particular, is predominantly identified in phylogroups A and B1, while EPEC is most often identified in phylogroups B2 and B1.

**Answer:**As per suggestion**,** phylogenetic classification has been corrected in the revised manuscript, and reference has been added (Ref. 22) (please refer lines 140-145).

**Question 41.**Lines 163-164: Please provide a reference for the Vesikari score.

**Answer:** As per suggestion, reference (Ref. 29) for the Vesikari score has been provided in the revised manuscript (please refer line 172).

**Question 42**. Line 189: Please describe the region amplified by the CVD432 primers.

**Answer:** Size of the pCVD432 gene is 765 bp and the primer utilized in this study amplified the region from start position 65 to the end position 694 and which makes the product size of 630 bp. The amplified region of pCVD432 gene has been highlighted below (please refer lines 211-213).

> pCVD432 sequence 765 bps ATCGAGTGAGACACATATTATACTCATTTCTTGCAATAAATGCTTATCTGTTTTCGACACAGACTCTGGCGAAAGACTGTATCATTGATAATTTCTTTCAGAAAAGCATCCAGTTTAATTCTTATTCTCTTGATATCGAAGAGTTAGATATTAATAAACATAACAATATAAAANCGATGTTACCAGATATAAATATAGGGTTAGGGCAGTATATAAACAACAATCAATGGTTCTCATCTATTACAGACAGCCATTTTTATTTATCATTATCCTATAATCTTCTATCGGCTTATGAAGCAAAAATGCAGAATAATAAATTGGATATTGCTAATTATTTAAAATATATTGAAATGCTTAGTGAGAGGAACAACTACATAATTAATTTGCTCTCGGAAATTATTAACTATAAGATAAAAAAATCTCACCTGATGTTGATGCTCGAGAGATATAGGAAGCTCAATAAAGAATACGAAATTGCAAAGCATAAAATGTCAATTGGATTAATATCTGCTCTTGATGTAGAGATGATATATAATATTTTACAAAAAATCAGGTTTGATATTGATGTCCTTGAGGAGGAGGAAAGTTTACTGTCAGATAAAATCTCGAGAGAATATCATGTTCCTGAGAGTGCAATCCCAGACATTACATATCATAAGTTAAAAGAGTGTAAAACAGCGGATTTCTATACATTATTAGCTGAAAACAAAAAACTCAAGATTAAGGCTGCTGATATAGATAATGATATAAGGAAACTATCTGAGA

**Question 43.** Lines 204-206: Were the aEAEC identified only by the absence of aggR? Or they also contained the CVD432 region? Were they verified phenotypically to be EAEC by their adherence patterns in HEp-2 cell culture?

**Answer:** All the aEAEC stains contained the CVD432 region, but they do not carry the *agg*R gene. No, they are not phenotypically tested by adherence patterns in HEp-2 cell culture.

**Question 44.** Line 242: This includes both tEAEC and aEAEC?

**Answer:** No, it includes all EAEC and other DEC pathotypes isolated from the diarrheal group (please refer lines 268-272).

**Question 45.** Lines 572-573: The sentence starting with “Many EAEC strains….” is not clear.

**Answer**: “Many EAEC strains” in line 572-573 mean that in our findings,  most of EAEC strains from the diarrheal group belong to B2 and D phylogroups, which are potentially pathogenic (please refer lines 575-576).

**Reviewer #2:**

This is a really extensive piece of work and must be shared with the scientific community. However, the data presentation needs to fine tuned. There is a lot of repetition. The paper should be made concise for better understanding.

Reviewer #3: (No Response)
